# Implementing Professional Midwife-Led Maternity Care in India for Healthy Pregnant Women: A Community Case Study

**DOI:** 10.3389/fpubh.2022.875595

**Published:** 2022-06-09

**Authors:** Andy Beckingham, Soo Downe, Evita Fernandez, Becky Reed, Indie Kaur, Nuzhat Aziz, Carol Kingdon

**Affiliations:** ^1^Fernandez Foundation, Hyderabad, India; ^2^UCLan THRIVE Centre, Research in Childbirth and Health (ReaCH) Unit, University of Central Lancashire, Preston, United Kingdom; ^3^Midwife, London, United Kingdom

**Keywords:** midwifery, LMICs, India, respectful maternity care, care continuity, midwifery education, woman-centered care

## Abstract

More women and neonates die each year in India than in almost every other country of the world. Since 1947, India has in principle provided free medical maternal health care to all pregnant and childbearing women. Although rates of maternal and neonatal deaths have fallen since the 1990s, major inequalities remain. Some Indian States have very high rates of interventions, (e.g., cesarean section), while others have intervention and care rates that are too low. Disrespectful treatment of women in labor and lack of evidence-based practice have also been reported. The World Health Organization and others have strongly recommended that professional midwives (trained in a woman-centered philosophy and to international standards) have a key role for reducing mortality and morbidity, minimizing unnecessary interventions in pregnancy and labor, and improving maternal care quality in low- and medium-income countries. This paper provides a community case-report of the first professional midwifery programme in India designed to international standards, implemented in 2011 in Hyderabad. We describe the design and implementation in the programme's first eleven years, as a basis for further scale-up and testing in India, and in other low- or medium-income countries. The ultimate aim is to improve maternal care quality, choice and outcomes in India and in similar socio-economic and cultural settings.

## Introduction

### The Problem Being Addressed

With a population of more than 1.3 billion people in 2022, and about a sixth of the world's population, India accounts for a significant part of the world's maternal morbidity, mortality and unmet health needs. Although maternal mortality declined since 2004-6 (1), the 2020 rate was still estimated as 99 maternal deaths per 100,000 live births (2). The overall burden of morbidity was even higher (3, 4). A range of community and health facility surveys suggest high rates of anemia, pregnancy-induced hypertension, eclampsia, hemorrhage, puerperal sepsis, obstructed labor, and high rates of post-delivery morbidities (5). The most disadvantaged women have the worst maternal health and treatment (6–10). India ranks 140 out of 156 on the world's Gender Inequality Index (11), and there is evidence of a general lack of regard for women's rights and views (12–14). Many maternity care quality improvements promoted by the national Government were not implemented by a significant proportion of care providers (15, 16). Widespread deficits in infrastructure, supplies, equipment, and staffing compound these problems (17, 18). Abuse, disrespect, mistreatment and lack of privacy in maternity care institutions have all been reported (17, 19–24). High rates of unnecessary interventions in labor and childbirth (notably cesarean section without clinical indication) have been recorded across the country's public and private maternity services, in parallel with very low rates in remote rural and more socio-economically disadvantaged urban areas (25). Despite substantial national Government promotion, uptake of evidence-based care has often been poor (24). Widespread deficiencies in woman-centered labor support have been documented (26).

Following Independence, the Indian Nursing Council merged nursing and midwifery, and the midwifery education elements diminished thereafter. The official model of maternity care in India in 2011 centered on obstetricians as lead providers, assisted by a cadre of “nurse/midwives” with low status, brief midwifery training and regarded generally as “an appendage of obstetrics” (27) working under the direction of doctors in institutional facilities. Many of India's public hospitals have insufficient numbers of doctors (28) to ensure effective management of maternity problems and support straightforward labor and childbirth.

### Rationale for the Innovation

The lack of a specific midwifery profession in India has been criticized (29–31). Countries that introduced midwifery have seen declines in their maternal mortality (32). There is strong evidence from systematic reviews that relationship-based midwife continuity of care through pregnancy, birth and the postnatal period is associated with reductions in adverse outcomes, reductions in unnecessary clinical and technical interventions in labor and birth, improved maternal wellbeing, and reduced health care costs, both for healthy women, and for those with complications (33). In 2014, the Lancet Series on Midwifery noted that midwifery, as defined by the Lancet Quality Maternal and Newborn Care (34) framework has the potential to prevent more than 80% of maternal and neonatal deaths globally, including stillbirths (33, 35, 36). The current World Health Organization guidelines for antenatal and for intrapartum care (37, 38) recommend the introduction of professional midwifery to improve the quality and outcomes of maternity care. The criteria for professional midwifery, established by the International Confederation of Midwives are based on good quality evidence, and respectful and relationship-based care provision (39).

## Context of The Innovation

The midwifery programme was established in 2011 in the Fernandez Hospital group, which provided private maternal and neonatal care in the Telangana state of India, but became a not-for-profit Foundation in August 2018. The main site was a 320-bed specialist tertiary teaching hospital providing care for more than 9000 women per year. The hospital was committed to high-quality evidence-based care and protocols, and to continuous clinical reviews. Until the advent of professional midwifery, 44 doctors and 130 nurses provided the care for all healthy childbearing women and those with complications (Note: staffing numbers are also Whole Time Equivalents. All midwives, doctors and nurses at the hospital are full time and work 48 h per week).

WHO and the ICM recommend that midwifery programmes be developed in an enabling environment, with good facilities, transport and communications, a respectful safe working environment, coordination of integrated care, mentorship and peer support, and professional and career development (40). Fernandez Hospital offered a quality-assured setting with immediate access to high-quality specialized obstetric and neonatal care throughout the piloting.

In 2010, the managing director of the hospitals group (EF, an obstetrician) proposed the midwifery pilot to the senior obstetricians. A brief epidemiological needs assessment plus a series of stakeholder interviews were undertaken by the hospital's UK public health consultant (AB, first author) in collaboration with the managing director, to identify Telangana's maternal health needs, inequalities and morbidity, in order to help assess the need for midwifery, This indicated a great deal of poor maternal health and care in many public and private hospitals in the State, where obstetricians often struggled to provide appropriate care for healthy women and babies while also trying to manage the care of higher risk women and emergencies. Telangana had one of the highest rates of cesarean section (CS) in the country (58% of births in 2016) (41, 42), while the maternal mortality rate was estimated at 81 per 100,000 live births per year in 2018-19 (43). Respectful care, privacy and choice for women were and still are often missing (44). Neglect and abuse were commonly reported. Globalization of the “birth market” (48) plus government payments to women to give birth in hospitals had resulted in a high-volume “industrialized” maternity system, and there was an overwhelming need to “humanize childbirth” (49) and provide good quality, woman-centered care and normal birth for those women without clinical indications for intervention in labor.

The managing director was familiar with the role of the midwife in the UK, where midwives are educated to international standards and enabled by legislation and policy to be autonomous practitioners, providing midwifery care for most women in the antenatal, intrapartum and postnatal periods, unless medical or obstetric problems arise. In the UK, obstetricians focus on the management of complex pregnancies. This was seen as a potentially useful model for the hospital group, so a midwifery education and training programme based on ICM competencies and on evidence-based and woman-centered care was designed and adapted in consultation with the managing director and with senior doctors.

The goal of the innovation was to develop and deploy a new autonomous profession of midwives, educated to provide woman-centered maternity care for healthy pregnant women, working collaboratively with doctors, and to test midwifery's acceptability in India.

## Key Programme Elements

### Design of the Education Programme

The Professional Midwifery Education and Training (PMET) was designed to equip a new midwifery profession that would satisfy the following criteria:

- Midwives meeting ICM competency criteria.
- Effective collaborative working between midwives and obstetricians.
- Improvements in women's experiences of care.
- Improved quality of maternity care, with particular relevance to:

° Respectful care.
° Women being given information and choice.
° Woman-centered care: challenge to institution-centered care.
° Skin-to-skin contact at birth and breastfeeding within the first hour of birth.
° Capacity to support women to achieve physiological birth if that is their choice.

Initially the plan had been to educate women with appropriate qualities and experience to become midwives *via* a 3-year programme that did not require a prior nursing qualification, but this could not be implemented because the hospital group was funding the project internally. Consequently, the programme was offered to qualified nurses as a postgraduate programme, comprising 18-months of formal midwifery education plus a further 6 months of supervised practice.

### Timeline for Implementation

Table 1 presents a time-line of all the key events in implementation (Each stage is also described narratively).

**Table 1:** Key events in the implementation of Professional Midwifery Education and Training in India, 2011-2019.

Jan 2011 Managing Director forms a steering group and engages the hospital's senior obstetricians in a pilot of midwifery
Mar 2011 Needs assessment conducted; Design of core principles, education programme and curriculum begins.
May 2011 Steering Group begins planning and recruitment.
Aug 2011 The first cohort of Professional Midwifery students begin their education and training on-the-job.
Dec 2011 The first UK senior midwife begins mentoring and teaching.
February 2013 The first cohort of professional midwives qualify and begin 6 months' supervised practice.
June 2017 National Government Minister with maternal health portfolio is briefed about the PMET initiative.
Oct 2017 Partnership formed between PMET, State Government and UNICEF to pilot PMET in two public hospitals. Thirty government nurses recruited to train and work as Professional Midwives.

### Project Management

A steering group for midwifery development was appointed, consisting initially of the MD, three senior obstetricians, the chief nurse and the public health consultant. Potential limitations were that no UK midwife was initially available, and service users with experience of midwifery care were not known of in the local population. Once the programme became established, the steering group continued to review clinical and quality aspects of the midwifery care. In 2017, a consultant midwife from the UK (IK, co-author) was appointed as director of midwifery and took over that remit.

### Design and Implementation of the Programme With the First Cohort

#### Programme Design

The programme was initially designed by AB, with online advice and support from senior, experienced UK-based midwives. It was informed by adult learning theories (45) in line with the contemporaneous midwifery education standards of the UK (46). The brief was to adapt a model that had achieved good outcomes to meet the health and care quality needs of an Indian population, and to design a curriculum based on it for Indian midwifery trainees. The Albany Midwifery Practice in London, UK, which had achieved exceptionally good outcomes among a very deprived population in London, was chosen as the index model.

The external validity of the programme was tested by ensuring that the curriculum was aligned to ICM competencies (47), and it was also critiqued against the curricula of two UK university midwifery degree courses. An experienced UK midwife (BR–co-author) joined the Steering Group, and played an active initial role in the iterative development of the curriculum and relevant printed and audio-visual materials, with emphasis on grounding it in woman-centered midwifery practice and values.

Refinement of the programme took place with the first cohort of students. After working with them in the classroom setting using the materials prepared as part of the programme development, BR acted as a role model and mentor, working with them in clinical practice to follow 25 women from admission to the labor ward to discharge home. This allowed them to observe and assist with exemplary, skilled, woman-centered midwifery care. These clinical practice sessions were followed by collective critical and reflective reviews of what they had observed, with reference to the principles and the evidence base of midwifery. The clinical engagement included learning at each birth how to assist women to mobilize during labor, how to explain choices about position, and how to facilitate delayed cord clamping, skin-to-skin contact and breastfeeding in the first hour. The managing director also organized sessions in which BR and a number of obstetricians discussed midwifery issues. The principles and concepts and clinical teaching came initially from the visiting UK midwives, grounding the trainees in midwifery as understood in the UK. But much of their “apprenticeship,” which developed their practice within an Indian culture took place through working with the senior Indian doctors, seeing how they interacted with the women. This was accompanied by clinical discussions and reflection led by the consultants.

#### Programme Content and Assessment Strategy

The key elements of the programme (content topics and teaching methods) are provided in Table 2.

**Table 2:** Key elements of the Professional Midwifery Education programme.

ICM Midwifery competencies
Philosophy and principles of midwifery
Scope of practice of Professional Midwives
Respectful maternity care, and women's choice
Principles and practice of woman-centered care
Continuity of midwife care
Curriculum, including:
Perspectives of midwifery practice
Physiological adaptation to pregnancy
Antenatal care
Screening during pregnancy
Reflective practice
Care of women in labor
Mobility and choice of positions in labor
Management of third stage
Importance of skin-to-skin contact at birth
Importance of breastfeeding within first hour of birth

The first cohort attended one full day's teaching/learning sessions per week for 18-months, and for the other 5 days of the week undertook clinical duties alongside the UK midwife tutor (BR). For subsequent cohorts, midwives who had graduated the programme took over this role, later led by the director of midwifery (IK). Teaching was provided in the hospital's academic suite, equipped with audio-visual aids and anatomical models. Digital material such as published evidence was distributed among the trainees, to read via their mobile phones. Presentations included slide shows, webinars, case studies, video presentations, and tutorials with themes, including critical appraisal of research papers on evidence-based theory and practice. All students received a copy of Myles Textbook for Midwives (50). The programme provided both direct (small group discussions, face- face interaction) and indirect teaching strategies (library use, use of internet sources for material). Trainees attended lectures and discussions, skills labs, objective structured clinical examinations, role play and drama, and journal clubs. Inter-professional learning and working was encouraged, while traditional rote learning (still common in India) was avoided.

The trainees' progress and performance were evaluated by continuous assessment over the eighteen-month course. They then received six group revision sessions, and took exams, conducted by in-house senior midwives and external examiners from the UK. Following graduation, they underwent 6 months of continually-assessed and supervised clinical practice. Those who were successful received certification and were offered contracts to undertake clinical work in a multidisciplinary setting at the Fernandez hospitals group as midwives. From 2017 this was done under the supervision of the director of midwives. Since 2011, for each year, a new recruitment round has been held and a new cohort of trainees have been enrolled.

#### Preparing the Local Staff

Most doctors and other staff in the hospital had not previously encountered midwives, and thus the project steering group expected some initial resistance to the idea that skilled midwives could provide maternity care for women with straightforward pregnancies. A change management programme was thus introduced, to optimize a positive reception for professional midwifery. The hospital's senior consultants were engaged by the MD in individual and group meetings to consider the prospect of piloting midwifery education and midwives' subsequent deployment at the hospital. They were asked to actively support the programme, promote it among their junior colleagues, and themselves advise on the education programme, curriculum and teaching plans. They also agreed to provide some of the teaching and to personally provide care jointly with the trainees. This had the additional benefit of showing the junior medical staff that senior consultants actively supported the programme. In 2018 when the midwives presented their annual outcomes statistics to an audience of doctors and nurses, the Head of Obstetrics stood and announced that she could no longer imagine working without midwives.

#### Selection Criteria and Recruitment to the Programme

Selection criteria and a job description were agreed incorporating key ICM competencies, and drawing on the UK Royal College of Midwives national job description for a midwife. Recruitment to the initial cohorts was from qualified local nurses with experience in maternity care, already employed by Fernandez Hospital. The hospital's MD (EF) personally conducted briefings for all eligible nurses, explaining what midwifery is, and how it is conducted in countries where it is mainstream. Candidates were also informed that because midwifery was such a new concept in India, their future careers as midwives could not be guaranteed outside the host training hospital.

Candidates needed a GNM nursing qualification plus 2 years' relevant work experience, a reference from the Head of Nursing stating that they had demonstrated a strong ethos of service to women, an ability to provide exemplary nursing care quality, and a high degree of empathy and kindness. They underwent a written examination plus formal interviews with a panel consisting of the MD, three consultant obstetricians and the public health consultant. Eight applied; all were successful.

### Development of the Programme Since 2011

The active engagement in practice and subsequent discussions that took place between the first cohort and their midwife mentor led them to critique the traditional separation of mother and neonates that took place routinely. Following negotiations with local neonatologists, this practice was changed to a new routine of keeping mother and baby together. This is one example of many that arose as the programme rolled out, which illustrate the power of midwifery philosophy when it is enacted in an organization that is open to change and continuous improvement. It was also an important learning issue, showing the trainees that midwives can influence change if they act with evidence and diplomacy.

For subsequent cohorts, additional UK midwives with a woman-centered approach were recruited to teach, train, mentor and conduct midwifery examinations. The trainees were still taught anatomy, physiology and related topics by obstetricians and neonatologists, becoming accustomed to multidisciplinary teaching. After 5 years, a UK consultant midwife was appointed as the programme's permanent Head of Midwifery (IK, co-author), to provide leadership and regular clinical supervision. In 2018 a full-time midwifery tutor was also engaged. During supervised training, each trainee has to support 40 women through labor and birth in order to qualify.

Over time, professional midwifery became systematically integrated into the hospital's work. An explanation of the role of Professional Midwives became part of the induction process for all new staff joining the hospital. Trainees, and later the qualified midwives, regularly took part in the hospital's weekly multidisciplinary reviews of inpatient cases, participated in drills and simulations on issues such as post-partum hemorrhage, and when qualified were encouraged to lead presentations for doctors, nurses and other midwives at the hospital. Case proformas had been used since the start of the programme for group reflective review among the trainees and tutor. The Midwifery Director, full-time tutor and the senior professional midwives developed the proformas further, conducted audits and care quality reviews, midwifery quality improvement becoming a continuous process.

In the early years of the programme, senior obstetricians had individually decided which women would be assigned to midwifery care. In 2018, a formal triaging system was introduced, in which senior midwives or consultants used a standard check list to book women into either midwifery, obstetric, or shared care.

The hospital is well-known for its educational events, which the MD has used systematically to influence care quality among clinicians, care providers and policy makers more widely in India. Professional midwifery trainees are routinely invited to all these workshops and conferences. The MD often speaks at international conferences, and after 3 years of the programme, qualified professional midwives sometimes acted as her co-presenters, later going on to present at international events alone, actively supported with material from the academic department, beingthus empowered to recognize their role as pioneers.

In the earliest years of PMET, trainee midwives had provided intra-partum and postpartum care, but not antenatal care. As the programme evolved and the graduate midwives gained more experience and themselves mentored trainees, a complete module of antenatal care was included in their education, and fully operationalised in 2017. Their antenatal work was initially supervised and assessed by senior obstetricians, until the Steering Group was satisfied that professional midwifery antenatal care was safe and should become a routine part of the hospital's service. It was henceforth supervised by the Midwifery Director and the senior professional midwives themselves.

### Programme Outcomes

Between August 2011 and December 2018, 67 midwives undertook the midwifery education course; 45 qualified, and 32 were still practicing, while 19 were still completing their training. Once qualified, each cohort of midwives mentored subsequent groups of trainees. In 2014 the hospital set as a target that all women with low-risk pregnancies should have their care provided by midwives, but this was not fully achieved as not enough midwives were available. However, by November 2019 two of the Fernandez Foundation hospitals had 70.2% and 86% of midwife-led births respectively, with a 66.7% Vaginal Birth After Cesarean (VBAC) rate. By March 2022, the trainees and graduate professional midwives had provided the maternity care for 15,895 women.

**Figure 1:**
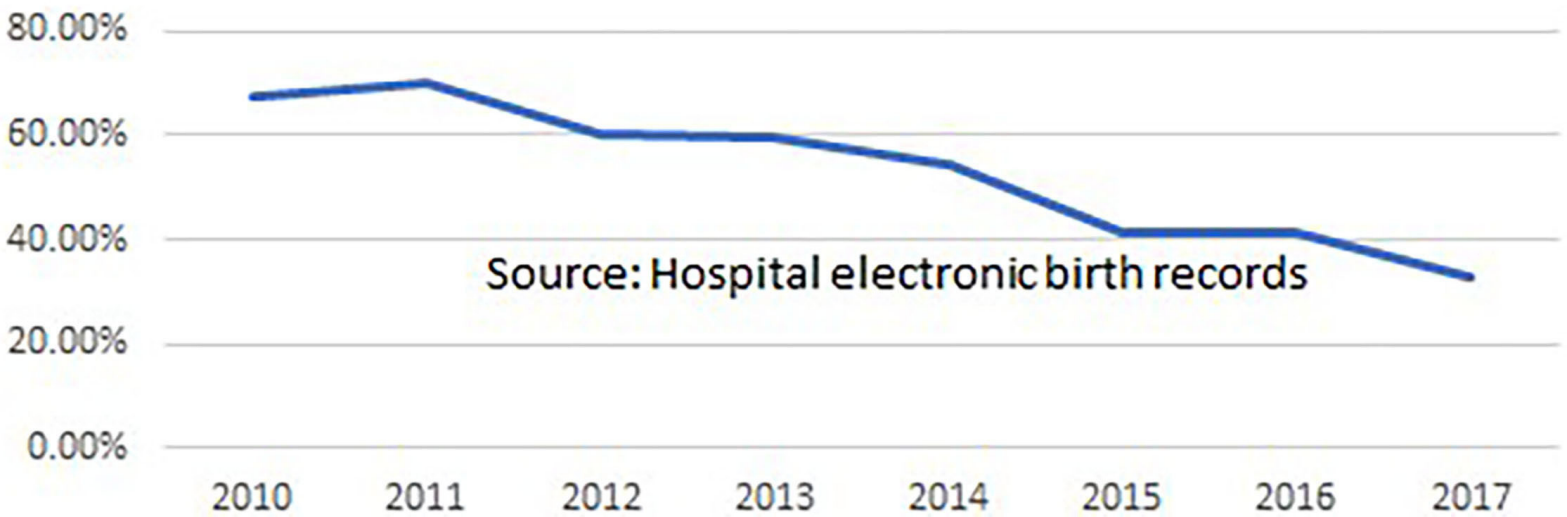
Epidural anesthesia rate for all women giving birth at Fernandez Hospitals, Dec 2010 to Dec 2017 (Professional Midwifery was implemented in Aug 2011).

**Figure 2:**
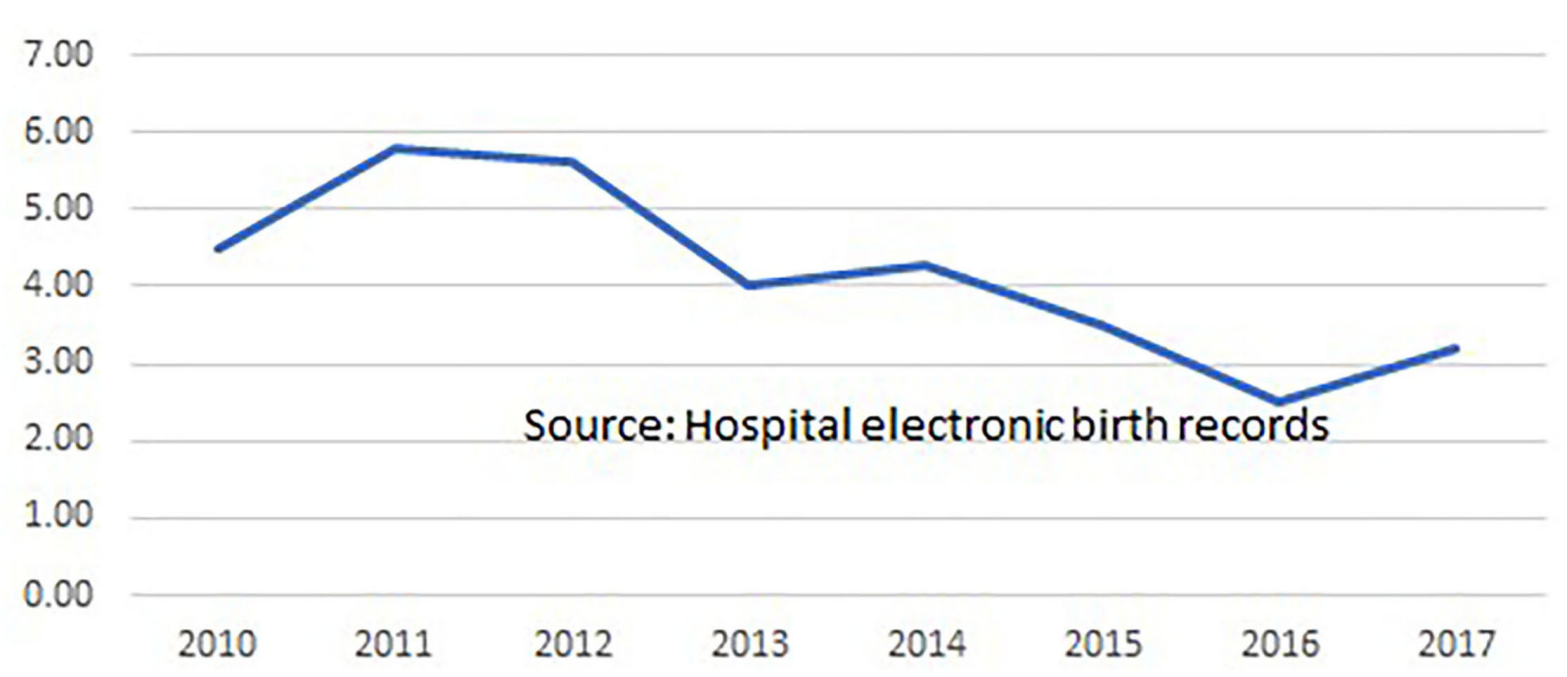
Stillbirth rate per 1000 booked singleton pregnancies, 28 weeks gestation and above, in Fernandez Hospitals, 2010 to 2017.

Clinicians at the hospital routinely record case notes for all admitted women, and a comprehensive set of data from every birth is extracted from these and transferred to the hospital's Electronic Medical Records database. In 2017, a proforma of midwifery data items (such as position in labor and birth, skin-to-skin contact and breastfeeding in the first hour) was added to the EMR data set. Evaluations of midwifery care quality and effectiveness were undertaken by the hospital's Research Fellow and other midwifery and medical staff. As shown in Figure 1 the hospital's overall epidural anesthesia rate declined from 70 to 32.8% during the period in which the midwives provided care. The episiotomy rate in the period 2007 to 2011 was 40%, falling to 27.4% in the period 2011 to 2022. It is not known whether or to what extent midwifery contributed to these improvements, but they would accord with evidence on midwifery care from around the world (33). Between August 2011 and March 2022, based on routine data collection on 15,895 births by spontaneous vaginal delivery supported by Professional Midwives and trainees, there was no increase in perinatal or maternal mortality or severe morbidity, or in third degree perineal tears or post-partum hemorrhage for women attended by midwives during labor and birth when compared to women with similar risk status attended by doctors. The episiotomy rate among women having SVDs attended by doctors (*n* = 21,741) was 21.1% while among those attended by Professional Midwives or trainees (*n* = 15,895) it was 6.3%. Figure 2 shows that the stillbirth rate at the hospital declined during the period 2011 to 2017.

A study at the hospital reporting on women's satisfaction with professional midwifery care, conducted via a routine survey at discharge, reported consistently high satisfaction levels over 3 years (51). Of 2,874 women whose labor and birth were assisted by professional midwives from 2015 to 2017, 2,486 (86.5%) completed forms, and 85.4% (2,123) of those responding reported their midwifery care as excellent. Rates of breastfeeding in the first hour of birth among women attended by Professional Midwives in the Foundation hospital and a number of public hospitals were consistently very high, and these will be reported elsewhere.

Over time many anecdotal reports were heard by the MD indicating that a reciprocal process of development may have taken place between the midwife trainees and the obstetric and neonatal educators and a number of the doctors who worked with the midwives, resulting in practice change, including active promotion of physiological birth. Annual surveys of the hospital's doctors about their views on midwifery will be reported separately but showed that over the first 3 years, the proportion of doctors expressing confidence in midwifery increased notably.

### Programme Replicability

The PMET programme was developed in a private (though not-for-profit) maternity care environment, so it was not known if public institutions might be willing to or capable of implementing it. However, in 2017, the hospital formed a partnership with the state's Ministry of Health & Family Welfare and UNICEF to pilot professional midwifery in a public hospital serving a disadvantaged population. The pilot was funded by the state government, and a Steering Group comprising senior personnel from the three organizations planned the development. Presentations were held for government nurses, explaining the concept of professional midwifery, and describing the personal qualities, skills and experience required for eligibility. Thirty qualified nurses were successful in their applications. They began their education at the same time as they began undertaking clinical work in the maternity unit at the public hospital, continuously supervised by senior midwives from the professional midwifery programme, and with 24/7 telephone access to midwifery support. The trainees completed a year's work at the public hospital in 2018 and then began 6 months of supervised practice at the programme's original hospital. Outcomes were continuously evaluated, again showing no adverse untoward events, while the outcomes of midwifery care appeared at least as good as those of other forms of care. These findings will be reported separately. In 2017, and now with support from the national Government, 30 new trainees from a number of other States have begun training at Fernandez Foundation as Professional Midwives and Educators.

## Discussion

The introduction of a new philosophical approach to midwifery in India was subject to a number of conceptual and social constraints. Medicine is a highly dominant profession in India, and, though most obstetricians are female, specific gender constraints still operate for midwifery, and may inhibit future recruitment. While it might also be seen by some to be problematic that a UK-type midwifery education was used as the basis for an India-focused programme, this was because notions of respectful woman-centered care are well established in the UK midwifery setting, and midwives are legally autonomous practitioners. Also, no woman-centered midwifery model could be identified within India.

The PMET programme was a pilot, since it was not known at the outset whether a woman-centered model of midwifery based on ICM standards and competencies would be transferable to an Indian setting. However, Professional Midwifery has now been sustained at the Foundation hospital for eleven years and has also shown significant potential in public hospital settings. More than 15,000 women with low-risk pregnancies in Telangana have had their maternity care provided safely and successfully by Professional Midwives. Hence while it was already known that midwifery is a highly effective form of maternity care for women in high-income countries, this article shows that this specific model of respectful woman-centered midwifery is safe, effective and culturally appropriate in India. Moreover, the clinical outcomes for women with low-risk pregnancies attended by the midwives were at least as good as for those women attended by obstetricians, and interventions tended to be lower. Professional Midwifery thus also appears to have potential to promote physiological birth and reduce unnecessary interventions in India.

Telangana state has a population less deprived overall than the average for India but still has millions living in poverty, plus one of the lowest rates of female literacy among Indian states – females 65.1%; males 80.5% (52). Many of the women attending Fernandez Hospital can pay for maternity care, but the hospital also provides care to many women in extreme poverty. Women attending speak a range of languages and are from Hindu, Muslim and many other cultures. Thus, while the population served by the midwifery pilot may not be as deprived overall as some others in India (53), the benefits might reasonably be expected if applied in many urban Indian populations. Further, this supposition is aided by the fact that Professional Midwifery has also been deployed successfully in a number of public hospitals in Telangana, serving a number of extremely disadvantaged communities.

### Lessons Learned for the Future

The success of this programme, and therefore lessons for roll out, include a range of factors. Local vision, leadership and commitment from an influential senior practitioner was essential in gaining the existing hospital workforce's support for the initiative, both for the education and to enable the qualified midwives to practice autonomously and effectively. A group of senior clinicians at the institution took an active part in the development, including teaching.

It was important that the programme took place in a setting with evidence-based protocols and continuous clinical review and improvement, and with a clinical and management culture open to challenge and willing to revise practice when presented with good evidence supporting it. This is in line with consensus about the key role of high-quality health systems in the attainment of Sustainable Development Goals (54).

It was also vital that the programme was based in a respectful, woman-centered, evidence-based philosophy of maternity care from the outset. The MD of Fernandez Hospital supported and advocated this approach throughout and it was reinforced by visiting senior UK midwives.

The programme benefitted from the allocation of sufficient funding for salaries of tutors, trainees, and midwives subsequently employed on graduation, and from voluntary contributions from senior experienced midwives with credentials in woman-centered care, curriculum, teaching and clinical leadership, from countries with well-established midwifery services, together with teaching by experienced senior graduate Professional Midwives.

## Data Availability Statement

The original contributions presented in the study are included in the article/supplementary material, further inquiries can be directed to the corresponding author/s.

## Author Contributions

EF initiated, led, funded, piloted, and hosted the PMET programme. AB drafted the PMET education programme and curriculum and planned and drafted this manuscript. AB, SD, and CK contributed to the detailed design and editing of the manuscript. NA provided and summarized hospital data for the manuscript. SD, EF, BR, and IK provided critical revisions for important intellectual content. All authors contributed to manuscript revision, read and approved the submitted version, and agree to be accountable for the work.

## Conflict of Interest

The authors declare that the research was conducted in the absence of any commercial or financial relationships that could be construed as a potential conflict of interest.

## Publisher's Note

All claims expressed in this article are solely those of the authors and do not necessarily represent those of their affiliated organizations, or those of the publisher, the editors and the reviewers. Any product that may be evaluated in this article, or claim that may be made by its manufacturer, is not guaranteed or endorsed by the publisher.
